# Repeated range expansion and niche shift in a volcanic hotspot archipelago: Radiation of C_4_ Hawaiian *Euphorbia* subgenus *Chamaesyce* (Euphorbiaceae)

**DOI:** 10.1002/ece3.4354

**Published:** 2018-07-30

**Authors:** Ya Yang, Clifford W. Morden, Margaret J. Sporck‐Koehler, Lawren Sack, Warren L. Wagner, Paul E. Berry

**Affiliations:** ^1^ Department of Ecology and Evolutionary Biology University of Michigan, Ann Arbor Ann Arbor Michigan; ^2^ Department of Botany University of Hawai`i at Mānoa Honolulu Hawai`i; ^3^ Division of Forestry & Wildlife Department of Land and Natural Resources Honolulu State of Hawai`i; ^4^ Department of Ecology and Evolutionary Biology University of California, Los Angeles Los Angeles California; ^5^ Smithsonian Institution Washington District of Columbia; ^6^ Department of Ecology and Evolutionary Biology University of Michigan Herbarium Ann Arbor Michigan; ^7^ Present address: Department of Plant and Microbial Biology University of Minnesota–Twin Cities Falcon Heights Minnesota USA

**Keywords:** *Euphorbia* subgenus *Chamaesyce*, Euphorbiaceae, Hawaiian Islands, section *Anisophyllum*

## Abstract

Woody perennial plants on islands have repeatedly evolved from herbaceous mainland ancestors. Although the majority of species in *Euphorbia* subgenus *Chamaesyce* section *Anisophyllum* (Euphorbiaceae) are small and herbaceous, a clade of 16 woody species diversified on the Hawaiian Islands. They are found in a broad range of habitats, including the only known C_4_ plants adapted to wet forest understories. We investigate the history of island colonization and habitat shift in this group. We sampled 153 individuals in 15 of the 16 native species of Hawaiian *Euphorbia* on six major Hawaiian Islands, plus 11 New World close relatives, to elucidate the biogeographic movement of this lineage within the Hawaiian island chain. We used a concatenated chloroplast DNA data set of more than eight kilobases in aligned length and applied maximum likelihood and Bayesian inference for phylogenetic reconstruction. Age and phylogeographic patterns were co‐estimated using BEAST. In addition, we used nuclear ribosomal ITS and the low‐copy genes *LEAFY* and *G3pdhC* to investigate the reticulate relationships within this radiation. Hawaiian *Euphorbia* first arrived on Kaua`i or Ni`ihau ca. 5 million years ago and subsequently diverged into 16 named species with extensive reticulation. During this process Hawaiian *Euphorbia* dispersed from older to younger islands through open vegetation that is disturbance‐prone. Species that occur under closed vegetation evolved *in situ* from open vegetation of the same island and are only found on the two oldest islands of Kaua`i and O`ahu. The biogeographic history of Hawaiian *Euphorbia* supports a progression rule with within‐island shifts from open to closed vegetation.

## 1 INTRODUCTION

Woody perennial plants have repeatedly evolved from herbaceous ancestors in isolated situations, such as islands and mountaintops (Bohle, Hilger, & Martin, 1996; Carlquist, 1974). The evolution of woody taxa from small, herbaceous mainland ancestors has occurred frequently on the Hawaiian Islands, the most remote island archipelago in the world (Carlquist, 1980). This phenomenon has been documented in a diversity of angiosperm lineages, such as the silversword alliance (Asteraceae, Baldwin, Kyhos, Dvorak, & Carr, 1991), violets (Violaceae, Ballard & Sytsma, 2000), *Plantago* (Plantaginaceae, Dunbar‐Co, Wieczorek, & Morden, 2008), *Silene* (Caryophyllaceae, Eggens, Popp, Nepokroeff, Wagner, & Oxelman, 2007), *Echium* (Boraginaceae, Bohle et al., 1996), *Schiedea* (Carlquist, 1995), and of note here, *Euphorbia* (Euphorbiaceae, Koutnik, 1987). Built by the successive emergence of volcanic islands, the Hawaiian Islands provide a natural system of time‐calibrated experiments of colonization and diversification (Lim & Marshall, 2017; Ziegler, 2002).

**Figure 1:**
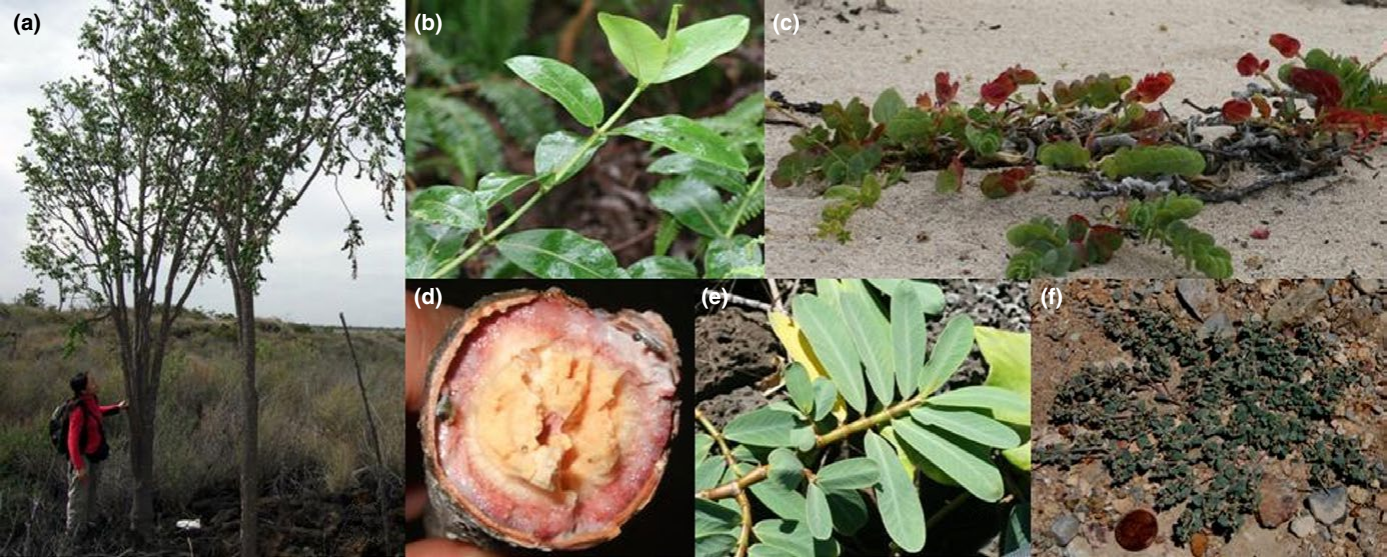
Hawaiian *Euphorbia* (a–e) and their closely related North American species (f). (a) *Euphorbia olowaluana*, a dry forest pioneer species on recently formed lava field, Hawai`i; (b) *E. remyi* var. *remyi*, an ascending shrub in wet forest understory, Kaua`i; (c) *E. degeneri*, a prostrate subshrub on sandy beach, O`ahu; (d) soft and fleshy woody stem of *E. celastroides* var. *kaenana*; (e) *E. celastroides* var. *kaenana*, a prostrate shrub, O`ahu; (f) *E. cinerascens*, a small, prostrate perennial herb native to deserts in southern United States and northeastern Mexico (see coin in the lower left corner for scale)

There are 17 *Euphorbia* species native to the Hawaiian Islands as recognized by the current morphologically based classification. One of them, *E. haeleeleana* belongs to *Euphorbia* subgenus *Euphorbia* (Dorsey et al., 2013). It represents a separate colonization event and is outside the scope of this study. The remaining 16 named species form a clade within *Euphorbia* subgenus *Chamaesyce* section *Anisophyllum*, hereafter referred to as Hawaiian *Euphorbia* (Yang & Berry, 2011). *Euphorbia* section *Anisophyllum* comprises about 400 species and mainly distributed in warm areas in North and South America (Halford & Harris, 2012; Yang et al., 2012). Members of the section are commonly small, weedy herbs, and all but three species exhibit C_4_ photosynthesis (Yang & Berry, 2011). Like other typical C_4_ plants, distribution of *Euphorbia* in the continental North and South America is mainly in warm, dry, and exposed habitats. In contrast, however, Hawaiian *Euphorbia* species occupy a wide variety of habitats, including coastal strand, dry forests, wet forests, and bogs, and they range in habit from subshrubs to trees 10 m tall (Figure 1). Four of the species have two or more recognized varieties. Ten species are endemic to a single major island, whereas the remainder is known from two or more major islands (Table 1). Six species and four varieties of Hawaiian *Euphorbia* are federally listed as endangered (marked with “*” in Table 1). A prior phylogenetic study with taxon sampling throughout section *Anisophyllum* suggested that Hawaiian *Euphorbia* originated following allopolyploidy, with their closest relatives being small herbs occurring in dry, warm, and exposed habitats in southern United States, northern Mexico, and the Caribbean, including *E. cinerascens*,* E*. *leucantha*,* E. mendezii*,* E. stictospora*, and *E. velleriflora* (Figure 1f; Yang & Berry, 2011). Given the overlapping distribution of these putative mainland close relatives, the allopolyploidy event likely happened before dispersal to the Hawaiian Islands. The long‐distance dispersal most likely occurred via the tiny seeds (typically 1–2 mm long) that adhere to birds with their mucilaginous seed coat (Carlquist, 1966, 1980; Price & Wagner, 2004).

**Table 1:** Distribution of the 16 named Hawaiian *Euphorbia* species on the six major Hawaiian Islands. Habitat types are sorted from wetter habitats generally at higher elevations to lower elevation and drier ones, and ages of islands are ordered left to right from older to younger (Koutnik, 1987; Koutnik & Huft, 1990; Lorence & Wagner, 1996; Morden & Gregoritza, 2005). Taxa with an “*” are federally listed as endangered. See Riina and Berry (2016) for species authorities

Species	Variety	Habit	Habitat	Kaua`i	O`ahu	Maui Nui			Hawai`i
						Moloka`i	Lana`i	Maui	
*sparsiflora*		Subshrub	Bog	X					
*remyi*	*hanaleiensis*	Shrub	Wet forest	X					
*remyi*	kauaiensis*	Shrub	Wet forest	X					
*remyi*	remyi*	Shrub	Wet forest	X					
rockii*		Shrub to small tree	Wet forest		X				
*clusiifolia*		Shrub	Mesic to wet forest		X				
halemanui*		Shrub	Mesic to wet forest	X					
*celastroides*	*hanapepensis*	Shrub	Mesic forest	X					
eleanoriae*		Shrub	Mesic forest	X					
*atrococca*		Shrub to small tree	Mesic forest	X					
herbstii*		Tree	Mesic forest		X				
deppeana*		Subshrub	Scrub to mesic forest		X				
*celastroides*	*tomentella*	Shrub	Dry to mesic forest		X				
*arnottiana*		Shrub	Dry to mesic forest		X			X	
*multiformis*	*multiformis*	Shrub	Dry to mesic forest		X			X	
*multiformis*	*microphylla*	Shrub	Dry to mesic forest	X	X	X		X	X
*olowaluana*		Tree	Dry forest and open sub‐alpine forest					X	X
*celastroides*	*amplectens*	Shrub	Dry forest	X	X	X	X	X	X
*celastroides*	*lorifolia*	Shrub to small tree	Dry forest				X	X	
*skottsbergii*	*vaccinioides*	Shrub	Scrub			X		X	
kuwaleana*		Shrub	Scrub		X				
*celastroides*	*celastroides*	Shrub	Coastal strand to dry forest	X					
*celastroides*	kaenana*	Shrub	Coastal strand to scrub		X				
*celastroides*	*laehiensis*	Shrub	Coastal strand to scrub				X	X	
*celastroides*	*stokesii*	Shrub	Coastal strand	X		X			
*degeneri*		Subshrub	Coastal strand	X	X	X		X	X
*skottsbergii*	*audens*	Shrub	Coastal strand			X			
*skottsbergii*	skottsbergii*	Shrub	Coastal strand		X				

Following their arrival on the Hawaiian Islands, Hawaiian *Euphorbia* became woody, and some species lost the mucilaginous seed coat and developed larger seeds (Carlquist, 1966). Yet all species retained C_4_ photosynthesis like their close mainland relatives (Pearcy & Troughton, 1975; Sporck, 2011). C_4_ photosynthesis is a specialized adaptation typically providing a competitive advantage under low CO_2_ availability and/or in hot, dry environments (Sage & McKown, 2006). By contrast, Hawaiian *Euphorbia* species such as *E. remyi* grow in wet forest understory under low light (Figure 1b). The Hawaiian *Euphorbia* is thus an interesting model group for understanding the evolution of photosynthetic systems (Sage & Sultmanis, 2016).

In this study, we sequenced seven chloroplast and three nuclear markers to reconstruct the history of radiation in Hawaiian *Euphorbia*. Specifically, we investigate the sequence of Hawaiian *Euphorbia* colonizing major islands along the Hawaiian island chain. We tested whether Hawaiian *Euphorbia* moved into forest understory a single time and then dispersed among islands, or if they moved into forest understory independently on different islands.

## 2 MATERIALS AND METHODS

### 2.1 Taxon sampling

A total of 153 Hawaiian DNA accessions representing 15 of the 16 species of Hawaiian *Euphorbia* were included in this study. A 16th species, *E. eleanoriae*, that belongs to the studied group was missing from our taxon sampling due to its remote location restricted to steep cliffs of Kaua`i (Lorence & Wagner, 1996). Although Hawaiian *Euphorbia* occurs on all major islands, our samples focused on six of the highest Hawaiian Islands: Kaua`i, O`ahu, Moloka`i, Maui, Lana`i and Hawai`i. The islands of Moloka`i, Maui, and Lana`i together form the Maui Nui island group, a reflection of their close proximity and past land connection as recent as the last interglacial period (Price & Elliott‐Fisk, 2004). Of the 153 DNA accessions, 125 were obtained from the Hawaiian Plant DNA Library (Morden, Caraway, & Motley, 1996; Randell & Morden, 1999), complemented by 18 additional samples newly collected from the field or cultivated sources (Supporting Information Appendix S1). Forty‐three DNA accessions in the DNA Library were collected by M.J. Sporck‐Koehler and L. Sack, accompanied by some of Hawaii's most experienced field botanists (see Acknowledgments) as part of an ecophysiological study of the C_4_ Hawaiian *Euphorbia* (Sporck, 2011); permits for many of the species were limited to less than ten leaves per plant, with vouchers not permitted for state and federally listed endangered or very rare and extremely vulnerable taxa. We describe in Supporting Information Appendix S1 locality information for source populations and alternative voucher specimens representing the same population.

The resulting infraspecific sampling ranged between 1 and 23 accessions per species. For species such as *E. deppeana*, found in only one wild population with ca. 50 individuals in total, only one accession was included; by contrast, for *E. celastroides* var. *amplectens* and *E. degeneri*, found on all major Hawaiian Islands, 12 and 13 accessions were included, respectively, representing multiple populations from different islands. To distinguish among different accessions of the same taxon, we included DNA accession numbers following taxon names for all the ingroup Hawaiian *Euphorbia* in the text. In addition, 11 closely related North American species were selected for outgroup comparison based on the previous comprehensive, section‐wide phylogenetic analysis of Yang and Berry (2011).

### 2.2 Laboratory procedures

Genomic DNA extraction, plus PCR amplification and sequencing of both ITS and cpDNA followed the protocols in Yang and Berry (2011). A total of seven chloroplast (cpDNA) noncoding regions were sequenced: *atpI*‐*atpH* spacer, *psbB*‐*psbH* spacer, *psbD*‐*trnT* spacer, *rpl14*‐*rpl36* spacer, *rpl16* intron, *trnH*‐*psbA* spacer, and the *trnL‐F* region. For the ITS region, sequences with continuous superimposed peaks were excluded. Two of these excluded PCR products, *E. celastroides* var. *kaenana* 5840 and *E. kuwaleana* 5700, were cloned following the protocol of Yang and Berry (2011) to evaluate allelic variation. The second intron of the nuclear low‐copy gene *LEAFY* and intron of *glyceraldehyde 3‐phosphate dehydrogenase subunit C* (*G3pdhC*) were PCR amplified and cloned following the protocol in Yang and Berry (2011), except that at least 24 clones from each PCR product were sequenced to recover all copies. Copy‐specific primer pairs were designed for both *LEAFY* and *G3pdhC*, and at least eight clones were sequenced from each copy‐specific PCR reaction (Supporting Information Methods in Appendix S2).

### 2.3 Phylogenetic analysis

Each of the seven cpDNA and three nuclear data sets were analyzed separately using maximum parsimony (MP) in PAUP* (Swofford, 2003). Heuristic searches were performed with 1,000 random addition replicates holding one tree per step and keeping best trees only, MaxTrees = 10,000, with TBR branching swapping algorithm and saving one tree per replicate. Clade support was assessed by 500 bootstrap replicates as implemented in PAUP* with the following search settings: keep best tree only, stepwise addition, swap best tree only, MaxTrees = 1,000, 1,000 random replications of sequence addition, holding one tree at each step, TBR branch swapping, and multitrees on. Preliminary MP analyses using individual cpDNA regions detected three short inversions (Supporting Information Methods, Appendix S2). The three inversions were reversed and complemented before concatenating all seven cpDNA regions into the first character set of the cpDNA matrix. Indels were scored following the simple gap‐coding criterion (Simmons & Ochoterena, 2000) in SeqState v1.4.1 (Müller, 2006) and were treated as the second character set of the cpDNA matrix.

Bayesian inference was conducted in MrBayes v3.1.2 (Huelsenbeck & Ronquist, 2001; Ronquist & Huelsenbeck, 2003). Two independent runs of four chains each (three heated, one cold), starting from random trees, using a temperature of 0.2, were run for 10 million generations, using the model GTR + I + γ selected by AIC in MrModeltest v2.3 (Nylander, 2004). Trees were sampled every 1,000 generations. Parameters were unlinked between the two partitions except tree topologies. The binary indels were subject to “rates=gamma.” A branch length prior “brlenspr=unconstrained:exponential(100.0)” was applied to the nucleotide partition to prevent unrealistically long branches (Marshall, 2010). Diagnostic parameters were visually examined in the program Tracer v1.5 (Rambaut & Drummond, 2007) to verify stationary status. Trees sampled from the first 1 million generations were discarded as burn‐in, and the remaining 18,002 trees were used to compute the majority rule consensus (MCC) tree and posterior probability (PP) for each branch of the MCC tree.

Maximum likelihood (ML) analysis was carried out using RAxML v7.4.2 (Stamatakis, 2006), partitioning nucleotides versus indels. The nucleotide substitution model was set to GTR + γ, and 500 rapid bootstrap (BS) replicates were performed, followed by a thorough search for the best tree.

### 2.4 Cross validation of date constraints and molecular dating using cpDNA

The Hawaiian island chain was formed by the Pacific plate moving northwestward over a fixed hot spot (Carson & Clague, 1995). We assumed that a new island was colonized soon after it emerged (Fleischer, Mcintosh, & Tarr, 1998), and that given the extremely small colonizing population, deep divergence from ancestral polymorphisms in the colonizing population was highly unlikely. We cross‐validated these two assumptions with a preliminary analysis estimating the stem ages of Maui Nui and Hawai`i clades by constraining the stem age of the oldest O`ahu‐based clade on the cpDNA data set with the time of full development of the Wai`anae Mountains (a normal prior with mean 3.86 million years [Myr] and standard deviation 0.089 Myr; Lerner, Meyer, James, Hofreiter, & Fleischer, 2011; Sherrod, Sinton, Watkins, & Brunt, 2007). A final analysis was carried out by applying the following age constraints to the cpDNA data set: (a) 3.86 ± 0.089 Myr for the stem age of the O`ahu‐based clade; and (b) 2.14 ± 0.117 Myr for the stem age of Maui Nui‐based clades (the age of Penguin Bank, which formed the past land connection between O`ahu and Maui Nui; Carson & Clague, 1995; Lerner et al., 2011; Price & Elliott‐Fisk, 2004; Sherrod et al., 2007). The analysis was performed in BEAST v1.7.4 (Drummond, Suchard, Xie, & Rambaut, 2012), using the concatenated cpDNA data set without coding indels. The substitution model HKY + I + γ was applied as selected by jModeltest v0.1.1 (Posada, 2008), with an uncorrelated lognormal relaxed clock and a pure‐birth Yule model. Four independent runs of 60 million generations were carried out, sampling every 10,000 generations starting from a random starting tree. Convergence diagnostic parameters were visualized in Tracer, and trees sampled from the first 6 million generations were discarded as burn‐in. A MCC tree was calculated in TreeAnnotator v1.7.4 (Drummond et al., 2012).

### 2.5 Phylogeographic reconstruction

Discrete phylogeographic analysis (Lemey, Rambaut, Drummond, & Suchard, 2009) was used to reconstruct the pattern of dispersal along the island chain from the cpDNA data set. Phylogeographic analysis was carried out in BEAST using two independent continuous‐time Markov chains by manually editing the xml file generated by BEAUti from the previous molecular dating analysis following Lemey et al. (2009). Most recent common ancestor of all Hawaiian accessions was set to Kaua`i according to molecular dating results. Convergence diagnostic parameters were visualized in Tracer, and the first 6 million generations were discarded as burn‐in.

### 2.6 Assignment of vegetation types

We categorized coastal strand, scrub, and dry forest habitats as “open vegetation.” Open vegetation is either fully exposed or has relatively open canopy coverage. It is generally low in elevation, though the upper elevation limit of lowland dry forest varies from 150 to 1,500 m depending on the island and the aspect of the slope, and the montane dry forests species *E. olowaluana* occurs in elevation as high as 2,800 m on Hawai`i (Gagné & Cuddihy, 1990; Koutnik & Huft, 1990). Both mesic and wet forests, which generally occur at relatively high elevation, have a closed forest canopy and were categorized as “closed vegetation.” Montane bogs, although not protected by a closed forest canopy, are specialized forest openings surrounded by wet or mesic forests and were categorized as closed vegetation.

## 3 RESULTS

### 3.1 cpDNA phylogeny and molecular dating suggested a Kaua`i/Ni`ihau origin of Hawaiian *Euphorbia*

**Figure 2:**
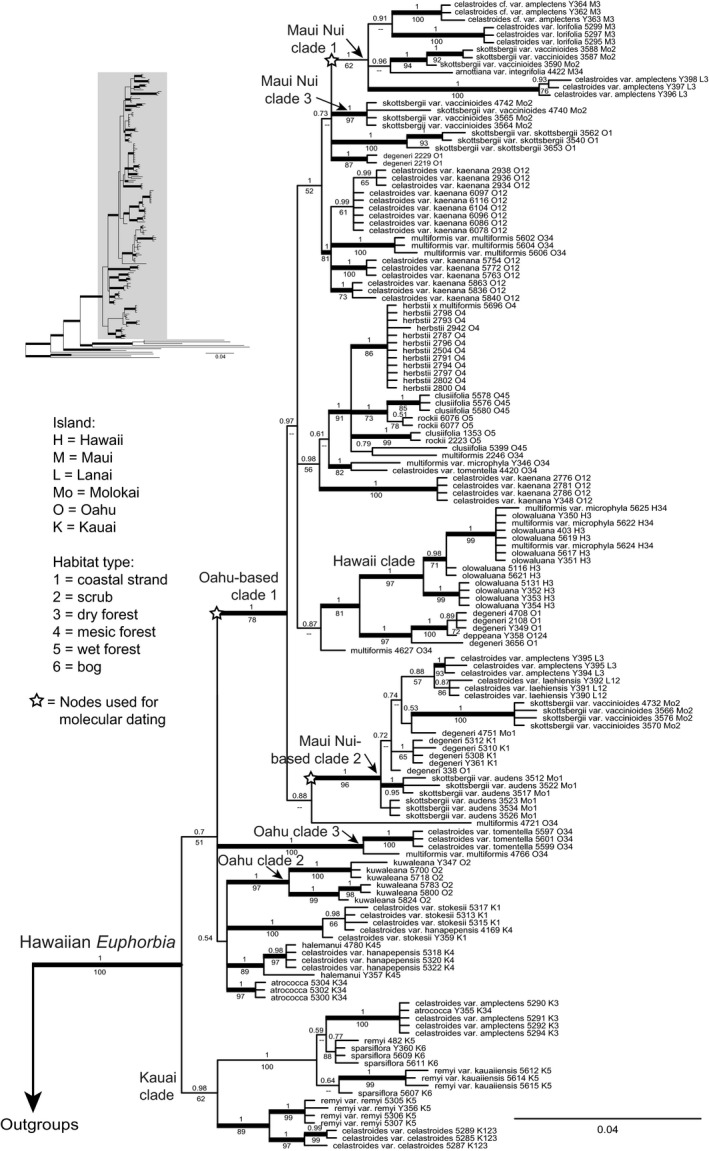
Majority rule consensus tree recovered from Bayesian analysis of cpDNA data in Hawaiian *Euphorbia*. Numbers above the branches are Bayesian posterior probabilities (PP) and numbers below the branches are maximum parsimony bootstrap percentages (BS). Branch length scale is on lower right. Thick branches represent strongly supported clades with PP ≥ 0.95 and BS ≥ 70. Outgroups were removed on the main graph, with the full tree in the upper left corner. Following each taxon name is the DNA accession number, island initials for the individual, and habitat type for each accession

We obtained sequences of all seven chloroplast noncoding regions from each of the 164 DNA accessions included in this study. The aligned matrix was 8,278 bp in length (alignment statistics in Supporting Information Table S2.1 in Appendix S2; alignment with inversions reversed and complemented in Supporting Information Appendix S3). Branch lengths within Hawaiian *Euphorbia* were much shorter compared to the outgroup species (Figure 2, upper left corner). Monophyly of Hawaiian *Euphorbia* was well supported (PP = 1 and BS = 100; Figure 2 & Supporting Information Figure S2.1 in Appendix S2). However, of the 13 species for which multiple individuals were represented in our sampling, 11 are either para‐ or polyphyletic according to the cpDNA tree, with the only exceptions being *E. herbstii* and *E. kuwaleana*, two rare species endemic to O`ahu (Figure 2). Despite being highly nonmonophyletic at the species level, the phylogeny displayed strong geographical structuring. A Kaua`i clade was sister to the rest of the Hawaiian *Euphorbia*, within which there are three well‐supported O`ahu‐based clades (PP = 1 and BS = 78, 97, and 100, respectively). Among the three O`ahu clades, the largest one (O`ahu‐based clade 1) had three well‐supported Maui Nui clades (PP = 1 and BS = 62, 96, and 97 respectively) and one well‐supported Hawai`i clade (PP = 1 and BS = 97) nested in it. The only Kaua`i members in the O`ahu‐based clade were a small clade of *E. degeneri* nested in Maui Nui‐based clade 2, which is a coastal strand species that occurs on all main islands.

We used cpDNA only for dating and phylogeographic analyses to track dispersal via seeds or vegetative fragments. Using island age for molecular dating can potentially be biased by delayed arrival long after island formation, multiple dispersal events, local extinction, and ancestral polymorphism. Another consideration is that at the time Kaua`i formed ca. 5 million years ago (Ma), the adjacent island of Ni`ihau was of similar size and prominence (Price & Clague, 2002). To cross‐validate our assumptions and their potential caveats, a preliminary analysis was carried out only constraining the stem age of O`ahu‐based clade 1, the most diverse and well supported O`ahu clade, by the date at which the Wai`anae Mountains of O`ahu formed (Figure 2; 3.86 ± 0.089 Myr; Lerner et al., 2011; Sherrod et al., 2007). The resulting estimate for the median stem age of Maui Nui clade 1 was 2.5 Myr (95% credibility interval 1.6–3.3 Myr), Maui Nui‐based clade 2 was 2.4 (1.5–3.2) Myr, and that for Maui Nui clade 3 was 1.4 (0.7–2.1) Myr. Both Maui Nui clades 1 and 2 had diversified on Maui Nui, and both had stem ages similar to the age of Maui Nui (ca. 2.1 Myr; Lerner et al., 2011; Sherrod et al., 2007). Maui Nui clade 3, on the other hand, is a much smaller and younger clade, including only a single coastal taxon and likely represents a more recent dispersal event. As for the Hawai`i clade, both its stem age (1.9 Myr; 1.1–2.9 Myr) and crown age (1.3 Myr; 0.7–2.1 Myr) were much older than the age of the island of Hawai`i (≈0.59 Myr; Lerner et al., 2011; Sherrod et al., 2007). Both taxa in the Hawai`i clade, *E. multiformis* var. *microphylla* and *E. olowaluana*, also occur on Maui Nui (Koutnik, 1987), and it is likely that the “Hawai`i clade” diverged on Maui Nui before dispersing to Hawai`i.

Based on our cross‐validation of dating points, our final molecular dating analysis constrained the stem age of O`ahu‐based clade 1 with the age of O`ahu (3.86 ± 0.089 Myr) and Maui Nui clade 1 and 2 with the age of Maui Nui (2.14 ± 0.117 Myr). The resulting stem age of Hawaiian *Euphorbia* was estimated at 5.0 (4.1–6.3) Myr, around the time that Kaua`i and Ni`ihau formed (ca. 5.1 Ma; Supporting Information Figure S2.2 in Appendix S2).

### 3.2 Phylogeographic reconstruction supports successive island colonization

**Figure 3:**
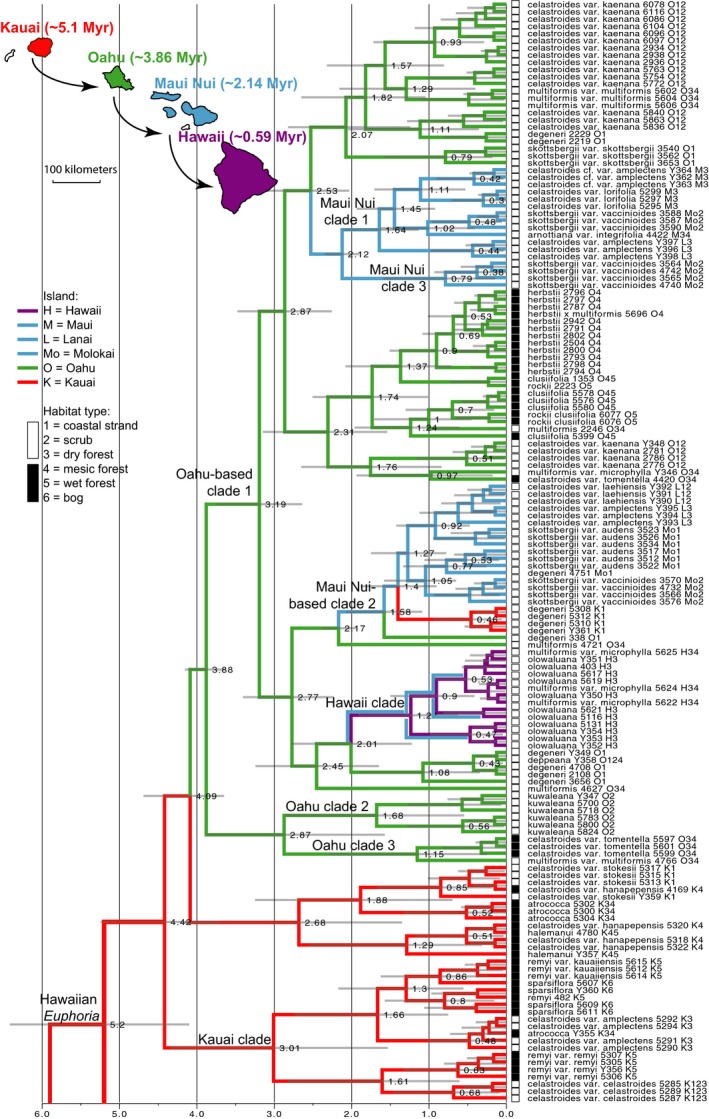
Maximum clade credibility (MCC) tree recovered from BEAST phylogeographic analysis in Hawaiian *Euphorbia*. Node labels are mean ages, and node bars are 95% highest posterior density (HPD) intervals. Outgroups are not shown. Following each taxon name is the DNA accession number, island initials for the individual, and habitat type for the taxon. Superimposed blue lines on the Hawai`i clade indicates the most likely scenario inferred from the clade age and historical distribution of *E. olowaluana* on Maui. Map in the upper left corner shows the inferred dominant pattern of dispersal among islands. Approximate age of each island is indicated on the map

By co‐estimating geographic distribution and tree topology, the resulting MCC tree from the phylogeographic reconstruction (Figure 3) very weakly supported O`ahu clades 1, 2, and 3 as monophyletic (PP = 0.46), as well as Maui Nui clades 1 and 3 as monophyletic (PP = 0.49), instead of being nonmonophyletic in phylogenetic analyses (Figure 2 & Supporting Information Figure S2.1 in Appendix S2) or the MCC tree of molecular dating alone (Supporting Information Figure S2.2 in Appendix S2). The stem age of Hawaiian *Euphorbia* is estimated to be 5.2 Myr (95% HPD 4.1–6.4 Myr). All analyses from cpDNA using RAxML, MrBayes, as well as molecular dating and phylogeographic reconstruction strongly support a general trend of successive island colonization from older to younger islands, despite the disagreements in weakly supported nodes.

### 3.3 Distribution of species richness across islands and habitats

**Figure 4:**
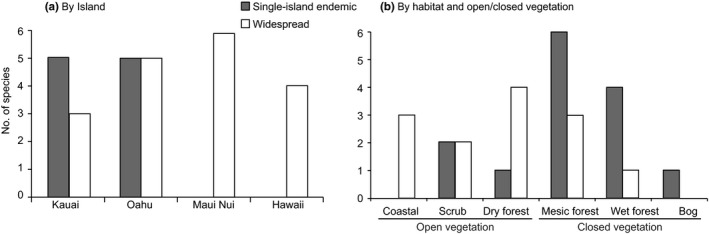
Distribution of Hawaiian *Euphorbia* species in (a) each major Hawaiian island, and (b) in each habitat and vegetation type

The number of overall species per island is highest in O`ahu (10 species), and decreases towards both older (eight on Kaua`i) and younger islands (six on Maui Nui and four on Hawai`i), showing a humped trend. Species that occupy two or more major islands (“widespread” species) were most numerous on Maui Nui, and single‐island endemic species were only found on Kaua`i and O`ahu, the two oldest islands, and absent from the two younger island groups (Figure 4a). The species‐habitat plot (Figure 4b) showed that “widespread” species tend to occur in open vegetation, while single‐island endemics tend to occur under closed vegetation.

### 3.4 All three nuclear markers had increased copy numbers compared to mainland relatives and low resolution within Hawaiian *Euphorbia*

**Figure 5:**
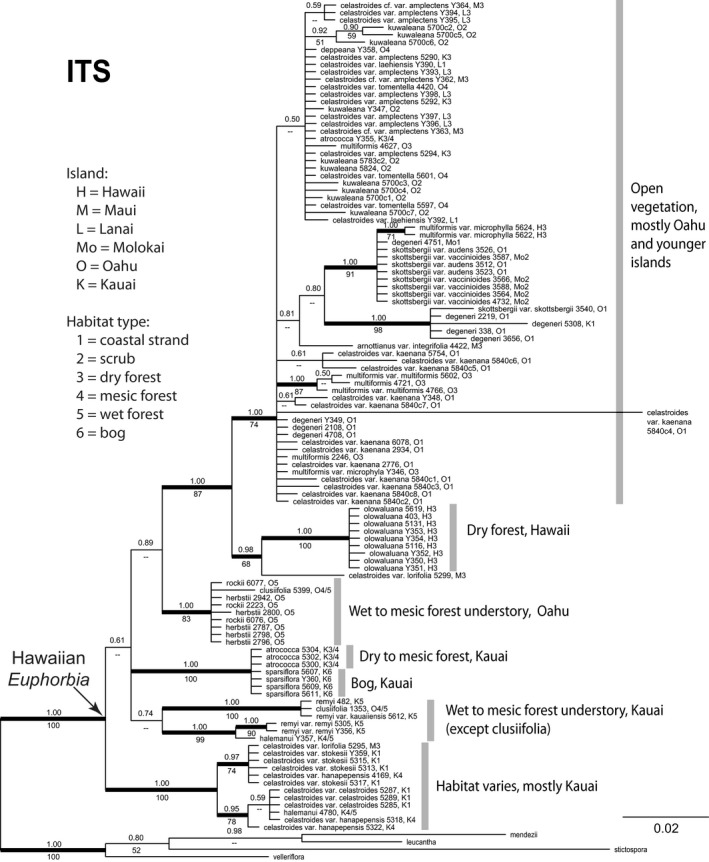
Majority rule consensus tree recovered from Bayesian analysis of ITS data in Hawaiian *Euphorbia*. Numbers above the branches are Bayesian posterior probabilities (PP) and numbers below the branches are maximum parsimony bootstrap percentages (BS). Branch length scale is on lower right. Thick branches represent strongly supported clades with PP ≥ 0.95 and BS ≥ 70. Following each taxon name is the DNA accession number, island initials for the individual, and habitat type for the accessions. Sequences from cloning of PCR produces have the clone number starts with “c” following the DNA accession number

Similar to the cpDNA phylogeny, the nuclear ITS tree highly supported the monophyly of Hawaiian *Euphorbia* (PP = 1 and BS = 100; Figure 5). All ingroup ITS sequences had 10 or more nucleotide positions showing superimposed peaks, which is much higher compared to outgroup taxa. In addition, 18 of the ingroup accessions showed continuously superimposed peaks, likely from allele length variation, and were excluded from the alignment. Cloning of *E. kuwaleana* 5700 and *E. celastroides* var. *kaenana* 5840 revealed many divergent alleles, including one on a very long branch (Figure 5). Although evolution of the ITS region was highly dynamic, there are nonetheless a number of well‐supported clades. Most of these clades occupied similar habitat types on a single island or open vegetation on O`ahu and younger islands (Figure 5).

Both the low‐copy nuclear genes *LEAFY* and *G3pdhC* showed increased copy numbers among Hawaiian taxa compared to outgroup taxa, but the resolution within each copy was low. Four copies of *LEAFY* were recovered, but only one copy was detected from the known outgroup species (Supporting Information Figure S2.3 in Appendix S2). Similarly, six copies of *G3pdhC* were detected in Hawaiian *Euphorbia*, among which three had a clear association with known outgroup species (Supporting Information Figure S2.4 in Appendix S2).

## 4 DISCUSSION

### 4.1 Kaua`i origin and dispersal following progression rule from older to younger islands

Our analyses suggest that Hawaiian *Euphorbia* first colonized Kaua`i or Ni`ihau, then O`ahu, Maui Nui, and finally Hawai`i, generally following the “progression rule” from older to younger islands (Funk & Wagner, 1995; Hennig, 1966), but with at least one dispersal event in the reverse direction through a widespread coastal species.

Our molecular dating analysis using ages of O`ahu and Maui Nui supported a Kaua`i or Ni`ihau origin of Hawaiian *Euphorbia*, given these two islands were of similar sizes 5 Ma (Price & Clague, 2002). The age estimation based on island formation is consistent with previous molecular dating analysis based on a Euphorbiaceae fossil and additional secondary calibration points, which estimated the split between *E. hirta* and *E. humifusa* to be ca. 9 Ma (Horn et al., 2014), a split much deeper than the stem of Hawaiian *Euphorbia* (Yang & Berry, 2011). Although our cross‐validating using island age largely corroborate with each other, such approach can potentially underestimate age of lineage diversification (Heads, 2011). On the other hand, secondary dating is known for its very broad confidence interval. The problem is further complicated by the molecular rate slow‐down associated with shifting from herbaceous plants on the mainland, to woody shrubs and trees in Hawaiian *Euphorbia*. In addition, the only reliable fossil suitable for molecular dating in Euphorbiaceae is outside of *Euphorbia*, and had a split with *Euphorbia* at approximately 75 mya (Horn et al., 2014). In order to constrain the root, Horn et al. (2014) used secondary dating points from the Malpighiales. With the broad taxonomic sampling Horn et al. (2014) used different markers than ours and we cannot directly combine data matrices from the two studies. To avoid tertiary dating, the most informative approach in our case is to take into consideration previous broad‐scale fossil dating in our discussion instead of attempting to carry out molecular dating using distantly related fossils.

Following the initial establishment in Kaua`i, dispersal from Kaua`i to O`ahu occurred at least once (Figure 3). There were at least two dispersal events from O`ahu to Maui Nui, followed by back‐dispersals from Maui Nui‐based clade 2 to Kaua`i and probably also to O`ahu, both involving the widespread coastal strand species *E. degeneri*. Although all individuals from Hawai`i form a monophyletic clade, given that its crown age (0.63–1.91 Myr) is significantly older than the age of the island (ca. 0.59 Myr) and that both *E. multiformis* var. *microphylla* and *E. olowaluana* also occur on Maui Nui, the Hawai`i clade likely split on Maui Nui before dispersing to Hawai`i, as indicated by the superimposed blue lines on Figure 3. Even though most species are nonmonophyletic in Hawaiian *Euphorbia*, given that we are using maternally inherited chloroplast regions for phylogeographic reconstruction, the biogeographic patterns we obtained are therefore tracing movement of the maternal lineage through either seeds or vegetative fragments.

### 4.2 Dispersal through open vegetation with *in situ* origin of species specialized in closed vegetation

Given that all closely related mainland species are from dry and disturbed habitats (Yang & Berry, 2011), the initial colonization of ancestral Hawaiian *Euphorbia* likely occurred in similarly open, disturbance‐prone vegetation on Kaua`i. Given that all species in open vegetation on Kaua`i are widespread and all species in closed vegetation on Kaua`i and O`ahu are single‐island endemics, the dispersal from Kaua`i to O`ahu likely also occurred through open vegetation. Species under closed vegetation that are generally in higher elevation (black squares in Figure 3) evolved independently on O`ahu versus Kaua`i, from open vegetation on the same island. A similar pattern of “upslope migration” is also evident in Hawaiian *Artemisia* (Hobbs & Baldwin, 2013) and in flightless alpine moths in Hawai`i and Maui (Medeiros & Gillespie, 2011). By contrast, in Hawaiian violets a nuclear ITS phylogeny recovered a “dry clade” and a “wet clade,” each having species from multiple islands (Havran, Sytsma, & Ballard, 2009). Given that Havran et al. (2009) relied solely on the ITS marker in a group with a complex polyploidy history, it may not have accurately resolved the evolutionary history of the group (Marcussen et al., 2012). Analyses of the Hawaiian endemic plant genus *Schiedea* using ITS + ETS + morphology (Wagner, Weller, & Sakai, 2005) and a more detailed assessment using eight plastid and three nuclear loci (Willyard et al., 2011) showed a pattern of multiple shifts to both dry and wet habitats from a presumed mesic ancestor.

### 4.3 Dynamic history of dispersal and habitat shift with island building and erosion

In addition to our findings of progressive dispersal along the island chain and movements toward closed habitats on individual islands, the timing of the volcanic island formation and erosion adds another dimension to the dynamics of dispersal and habitat shift (Lim & Marshall, 2017; Whittaker, Triantis, & Ladle, 2008). This is evident from the hump‐shaped curve of the total species number versus island age relationship typical in volcanic island systems (Lim & Marshall, 2017; Whittaker et al., 2008), here showing a peak on O`ahu (Figure 4a, adding dark and white). All single island endemic species occur on Kaua`i or O`ahu (Figure 4a), the two older islands, with most occur under closed vegetation (Figure 4b). Species that occur on more than one island can be found on any island (Figure 4a) and tend to occur in open vegetation (Figure 4b). These widespread species show a hump‐shaped distribution among islands, and their numbers peak on Maui Nui. No single‐island endemic species occur on Maui or Hawai`i, despite their current larger sizes and higher elevations than the older islands. Therefore it appears that when a young island emerges, it is first colonized by widespread species in open vegetation; and single‐island endemic species only arise later *in situ* through adaptation to forest understories, contributing to further increase of overall species number. As islands become older and eroded, the number of overall species decreases.

Both dispersal ability and habitat specialization in Hawaiian *Euphorbia* appear to be associated with seed characters. Hawaiian *Euphorbia* most likely arrived from North America via tiny seeds that adhered to birds through a mucilaginous seed coat (Carlquist, 1966, 1980; Price & Wagner, 2004). A survey of mucilaginous seed coats across *Euphorbia* sect. *Anisophyllum* (Jordan & Hayden, 1992) showed that it is present in most mainland species as well as in *E. celastroides*, one of the most widespread members of Hawaiian *Euphorbia*. The mucilaginous seed coat is absent, however, in all four single‐island endemic species surveyed: *E. clusiifolia*,* E. halemanui*,* E. remyi*, and *E. rockii*. Interestingly, *E. degeneri*, a widespread open vegetation species occurring on coastal strand of all major Hawaiian Islands, also lacks a mucilaginous seed coat. Instead, it is able to float in sea water (Carlquist, 1980), which likely explains its coastal distribution and offers an alternative dispersal mechanism besides sticking to birds. In contrast, neither *E. celastroides* (widespread) nor *E. clusiifolia* (Kaua`i endemic) appear to have floating seeds (Carlquist, 1966). In addition to the difference in dispersal ability between species of different vegetation types, endemic species, such as *E. clusiifolia* and *E. rockii* have seeds 2–3 times larger in diameter compared to typical widespread species (Koutnik, 1987). Such larger, nonsticky, nonbuoyant seeds may have enhanced seedling survival in forest understory with reduced dispersal ability.

### 4.4 Radiation of Hawaiian *Euphorbia* with gene tree nonmonophyly and extensive discordance between cpDNA and nuclear ITS markers

Our results from three nuclear markers supported the results from a previous analysis (Yang & Berry, 2011) that Hawaiian *Euphorbia* originated from a single colonization following allopolyploidy. Previous results from cloning another nuclear low‐copy gene, *EMB2765*, found three copies in Hawaiian *Euphorbia*. Two of the copies were associated with different mainland lineages, while a third copy had close relatives unresolved. With increased taxon sampling in this study, both nuclear low‐copy genes cloned, *LEAFY* and *G3pdhC*, also had increased copy numbers in Hawaiian *Euphorbia* compared to mainland species. Two of the four copies detected in *LEAFY* and three of the six copies detected in *G3pdhC* were not associated with outgroup taxa previously identified using ITS and chloroplast markers. In addition to the increased copy numbers in nuclear low‐copy genes, the elevated number of superimposed peaks recovered in the nuclear ribosomal ITS region compared to mainland relatives is also consistent with an allopolyploid ancestor for the Hawaiian *Euphorbia*.

Following arrival at the Hawaiian Islands, Hawaiian *Euphorbia* diversified with extensive gene tree nonmonophyly. Most species that occur in open vegetation are highly polyphyletic according to cpDNA (Figures 2 and 3). For example, *Euphorbia degeneri* is restricted to coastal beach habitats and is characterized by distinctive round and upwardly folded sessile leaves (Figure 1c; Koutnik, 1987). We included multiple Kaua`i, O`ahu, and Maui Nui accessions of *E. degeneri*, and they are placed by cpDNA in separate clades within O`ahu‐based clade 1 (Figures 2 and 3), and by ITS in a polytomy consisting mostly of open vegetation on O`ahu and younger island accessions (Figure 5). A second highly polyphyletic species, *E. celastroides* (Figure 1d–e), is variable in morphology and habitats and has eight recognized varieties (Table 1). Varieties of *E. celastroides* can be prostrate or upright, with leaf surfaces varying from glabrous to papillate, and the cyathia range from solitary to multiple. Each variety occupies one or more habitat types, from coastal strand to mesic forest, and may be either endemic to a single island or else more widespread. Notably, *E. celastroides* var. *kaenana*, which is endemic to the northwestern corner of O`ahu, is nonetheless polyphyletic and shows intermixture with *E. multiformis* from the same island in the cpDNA phylogeny (Figures 2 and 3), whereas ITS places all accessions of *E. celastroides* var. *kaenana* in a polytomy with species occupying open vegetation on O`ahu and younger islands (Figure 5). Despite being highly variable, *E. celastroides* is still morphologically distinctive, with entire, distichous leaves that are oblong to obovate in shape (Figure 1e). It can be distinguished from the vegetatively similar *E. multiformis*, also a widespread species, by its erect fruits and appressed cyathial glands, as opposed to recurved fruits and protruding glands in *E. multiformis* (Koutnik, 1985).

In addition to highly nonmonophyletic gene trees with deeply divergent placements, we also found evidence for more recent hybridization events. *Euphorbia multiformis* var. *microphylla* 5622 and 5624 were both collected at the Pohakuloa Training Area of Hawai`i, and they share an almost identical cpDNA haplotype with *E. olowaluana* accessions from the same area (Figures 2 and 3). In the ITS phylogeny, however, neither *E. multiformis* var. *microphylla* 5622 nor 5624 were grouped with *E. olowaluana*, but rather form part of a polytomy with other O`ahu and Maui Nui species that occupy open vegetation (Figure 5). Similar patterns of gene tree nonmonophyly are also found in other endemic Hawaiian plant lineages when multiple accessions were sampled. These include *Scaevola* (Goodeniaceae; Howarth & Baum, 2005), *Plantago* (Plantaginaceae; Dunbar‐Co et al., 2008), *Metrosideros* (Myrtaceae; Percy et al., 2008), *Pittosporum* (Pittosporaceae; Bacon, Allan, Zimmer, & Wagner, 2011), and *Bidens* (Asteraceae; Knope, Morden, Funk, & Fukami, 2012). Together these examples caution against using single representative samples per species, or relying on just cpDNA and/or ITS as the sole source for studying rapid radiations.

Certain infraspecific taxa in Hawaiian *Euphorbia* are geographically and morphologically distinctive enough that it is sometimes unclear whether separate species should be recognized (Koutnik, 1985, 1987; Koutnik & Huft, 1990). We decided not to recircumscribe species based on our results. First, some of the most morphologically homogenous taxa, such as *E. degeneri* and *E. celastroides* var. *kaenana*, are also some of the most polyphyletic. Second, with the highly dynamic allelic variation and low resolution in ITS, we do not have sufficient information to reconcile the discordance between ITS and cpDNA. Given that with seven cpDNA markers we had only moderate support for the overall relationships in Hawaiian *Euphorbia*, it will require high‐throughput sequencing with a larger number of additional markers to tease apart incomplete lineage sorting and ancient and/or recent hybridization as factors contributing to the tangled relationships among the Hawaiian *Euphorbia* species.

## 5 CONCLUSIONS

Our analyses of chloroplast regions suggest that after initial colonization of Kaua`i or Ni`ihau, Hawaiian *Euphorbia* moved from older to younger islands through dry and disturbed open vegetation, and species occupying closed vegetation evolved *in situ* on the older islands of Kaua`i and O`ahu. With recent and rapid divergence, many of the species as presently delimited show extensive nonmonophyly. The allopolyploidy origin of Hawaiian *Euphorbia* further complicates sequence analysis and leads to lack of clarity of the nuclear history.

## CONFLICT OF INTEREST

None declared.

## AUTHOR CONTRIBUTIONS

C.W.M., M.J.S., L.S., P.E.B., W.L.W, and Y.Y. conceived the ideas; C.W.M., M.J.S., L.S., and Y.Y. conducted the fieldwork; Y.Y. and C.W.M. carried out the lab work; Y.Y. analyzed the data; and Y.Y. led the writing.

## DATA ACCESSIBILITY

DNA sequence data were deposited in GenBank (Supporting Information Appendix S1). Alignment files in NEXUS format are provided in Supporting Information Appendix S3.
