# Supplementary material for: Repeated range expansion and niche shift in a volcanic hotspot archipelago: Radiation of C4 Hawaiian Euphorbia subgenus Chamaesyce (Euphorbiaceae)
Source: Ecol Evol. 2018 Jul 30;8(16):8523–36. doi: 10.1002/ece3.4354 (PMC6145001; doi:10.1002/ece3.4354)
Supplement: Supplementary file 2 [file ECE3-8-8523-s002.docx]

**Supplementary Methods**

**Primers used for PCR and Sanger sequencing.**

| Marker | Primers | Source |
| --- | --- | --- |
| ITS | ITS-I | Urbatsch et al., 2000 |
|  | ITS4 | White et al., 1990 |
| *rpl14*-*rpl36* spacer | rpL14 and rpL36 | Shaw *et al.*, 2007 |
| *psbB*-*psbH* spacer | psbB and psbH | Shaw *et al.*, 2005 |
| *atpI*-*atpH* spacer | atpI and atpH | Shaw *et al.*, 2007 |
| *psbD*-*trnT* spacer | psbD and trnT^(GGU)^-R | Shaw *et al.*, 2007 |
|  | Internal sequencing primer psbD-trnT F881 | 5’ TTG ATC TTG CGT TCT GGA ATC 3’ (designed in this study) |
|  | Internal sequencing primer psbD-trnT R1138 | 5’ CCT AAC CTA TTG CAT GAT GAC 3’ (designed in this study) |
| *trnH*-*psbA* spacer | trnH (GUG) and psbA | Hamilton, 1999 |
| *rpl16* intron | rpl16-71f | Jordan *et al*., 1996 |
|  | rpl16-1516r | Kelchner & Clark, 1997 |
| *trnL-F* region (amplified and sequenced in two pieces) | *trnL* intron: trnL-c and trnL-d | Taberlet et al., 1991 |
|  | *trnL-F* spacer: trnL-e and trnL-f | Taberlet et al., 1991 |
| *LEAFY* second intron | LFY F2 and LFY R1 | Howarth & Baum, 2005 |
|  | LFY1 F629 | 5’ TTC AGA CAC CTT TTG GGT T 3’  (designed in this study) |
|  | LFY1 R1415 | 5’ CTC GAC TTG ATT AGC ATA TTC TTG G 3’  (designed in this study) |
|  | LFY2 F177 | 5’ GGG TCC ACA GTA TAC CTA CCT AC 3’  (designed in this study) |
|  | LFY2 R1415 | 5’ CCA ACA TGA TTA GCA TAT TCC TGC 3’  (designed in this study) |
| *G3pdhC* intron | GPDX7F and GPDX9R | Strand et al., 1997 |
|  | GDX7 1F59 | 5’ TTC ACG CCA TCA CTG GTT AGT C 3’  (designed in this study) |
|  | GDX7 1R900 | 5’ TTA GGT TTC AGC AAG AGA ATC 3’  (designed in this study) |
|  | GDX7 2F144 | 5’ CTC CTT TGA ACT TGT GAT ACT G 3’  (designed in this study) |
|  | GDX7 2R850 | 5’ CAG YAA CAG AAA TGC TAA TGC CC 3’  (designed in this study) |

**PCR and cloning procedures using copy-specific primers of *LEAFY* and *G3pdhC***

Each PCR reaction contained 0.2 μL PfuUltra II Fusion HS DNA Polymerase (Agilent Technologies, Inc., Santa Clara, California, USA), 1.5 μL 10x buffer, 1.5 μL dNTP mix (2.5 mmol/L), 0.5 μL of each primer (10 μmol/L), 3 μL diluted template DNA, and ddH_2_O for a final volume of 15 μL. Cycling conditions were: 94 °C for 4 min; 35 cycles of 95 °C for 20 s, 51°C for 20 s, 72 °C for 1 min; and a final extension step of 72 °C for 3 min. PCR products were purified using the QIAquick PCR Purification Kit or the QIAquick Gel Extraction Kit. Purified PCR products were cloned using Zero Blunt TOPO PCR cloning kit (Invitrogen, Carlsbad, California, USA)

**Processing short inversions in cpDNA**

Two short inversions were detected in the *rpl16* intron region from a preliminary phylogenetic analysis using *rpl16* intron alone. The first one was a 33-bp inversion found in two outgroup accessions (*E. hirta* and *E. cinerascens*) and in six ingroup individuals (*E. remyi* var*. remyi* 5305 and Y356, *E. celastroides* var*. hanapepensis* 4169, *E. multiformis* var. *multiformis* 4766, *E. celastroides* var. *tomentella* 5597, and *E. celastroides* var. *stokesii* 5315). Monophyly of these eight individuals was strongly rejected by all other cpDNA markers. The second inversion in *rpl16* intron was 38 bp long and was found in *E. stictospora*, *E. velleriflora*, and *E. mendezii*. Monophyly of these three individuals was strongly supported by all cpDNA regions. A third 23-bp long cpDNA inversion was detected in the *trnH-psbA* spacer and was present in two outgroup individuals (*E. setosa* and *E. linguiformis*) and the ingroup *E. celastroides* var. *amplectens* Y396. Monophyly of these three individuals was again strongly rejected by all other cpDNA markers. All three short inversions were reversed and complimented in the final matrix for all subsequent analyses.

**Table S2.1 Alignment statistics**

**a. cpDNA regions**

| **Data set** | ***rpl16*** | ***trnH-psbA*** | ***trnL-F*** | ***rpl14-rpl36*** | ***psbB-psbH*** | ***atpI-atpH*** | ***psbD-trnT*** | **Concatenated cpDNA** |
| --- | --- | --- | --- | --- | --- | --- | --- | --- |
| **No. terminals** | 164 | 164 | 164 | 164 | 164 | 164 | 164 | 164 |
| **Aligned length** | 1432 | 904 | 1047 | 1007 | 613 | 993 | 2282 | 8278 |
| **Variable characters (proportion)** | 93 (6.5%) | 84 (9.3%) | 92 (8.8%) | 73 (7.2%) | 23 (3.8%) | 63 (6.3%) | 205 (9.0%) | 633 (7.6%) |
| **No. indels coded** | 41 | 31 | 60 | 17 | 7 | 40 | 94 | 292 |
| **Nucleotide substitution model selected by AIC** | – | – | – | – | – | – | – | GTR+I+γ |

**b. Nuclear regions**

| **Data set** | ***ITS*** | ***LFY*** | ***G3pdhC copy 1*** | ***G3pdhC copy 2-4*** | ***G3pdhC copy 5-6*** |
| --- | --- | --- | --- | --- | --- |
| **No. terminals** | 116 | 90 | 23 | 52 | 5 |
| **Aligned length** | 706 | 1447 | 1063 | 1030 | 929 |
| **Variable characters (proportion)** | 177 (25.1%) | 405 (29.2%) | 126 (11.9%) | 171 (16.6%) | 148 (15.9%) |
| **No. indels coded** | – | 88 | 12 | 19 | 12 |
| **Nucleotide substitution model selected by AIC** | GTR+I+γ | GTR+I+γ | – | – | – |

**Figure S2.1** Best tree from Maximum Likelihood analysis of cpDNA + indel in Hawaiian *Euphorbia*

**

**Figure S2.2** Maximum clade credibility tree recovered from molecular dating analysis of Hawaiian *Euphorbia*. Node labels are median age, and node bars are 95% highest posterior density interval.

**Figure S2.3** Majority rule consensus tree recovered from Bayesian analysis of *LEAFY* in Hawaiian *Euphorbia*. Numbers above the branches are Bayesian posterior probabilities and numbers below the branches are maximum parsimony bootstrap percentages. Branch length scale is on lower right. Following each taxon name is the DNA accession number and clone number when applicable. Copy-specific primer pair LFY2 F177 and LFY2 R1415 amplified copies 1 and 2, and primer pair LFY1 F629 and LFY1 R1415 amplified copies 3 and 4.

**Figure S2.4** Majority rule consensus tree recovered from maximum parsimony analysis of *G3pdhC* in Hawaiian *Euphorbia*. Numbers above the branches are bootstrap percentages. Following each taxon name is the DNA accession number, clone number when applicable, island initials, and vegetation type (see Fig. S2.3 for abbreviations). Copy-specific primer pair GDX7 1F59 and GDX7 1R900 amplified copy 1; primer pair GDX7 2F144 and GDX7 2R850 preferentially amplified copies 2 and 3; while copies 4, 5 and 6 were obtained from cloning PCR products from the original primer pair GPDX7F and GPDX9R. Only one tree was recovered from copies 5 and 6 and therefore the maximum parsimony phylogram is shown, with branch length scale on lower right.

**REFERENCES**

Hamilton, M.B. (1999) Four primer pairs for the amplification of chloroplast intergenic regions with intraspecific variation. *Molecular Ecology*, **8**, 521–523

Howarth, D.G. & Baum, D.A. (2005) Genealogical evidence of homoploid hybrid speciation in an adaptive radiation of *Scaevola* (goodeniaceae) in the Hawaiian Islands. *Evolution*, **59**, 948–961

Jordan, W.C., Courtney, M.W. & Neigel, J.E. (1996) Low levels of intraspecific genetic variation at a rapidly evolving chloroplast DNA locus in North American duckweeds (Lemnaceae). *American Journal of Botany*, 83, 430–439

Kelchner, S.A. & Clark, L.G. (1997) Molecular evolution and phylogenetic utility of the chloroplast *rpl16* intron in *Chusquea* and the Bambusoideae (Poaceae). *Molecular Phylogenetics and Evolution*, 8, 385­–397

Shaw, J., Lickey, E.B., Beck, J.T., Farmer, S.B., Liu, W.S., Miller, J., Siripun, K.C., Winder, C.T., Schilling, E.E. & Small, R.L. (2005) The tortoise and the hare II: Relative utility of 21 noncoding chloroplast DNA sequences for phylogenetic analysis. *American Journal of Botany*, **92**, 142–166

Shaw, J., Lickey, E.B., Schilling, E.E. & Small, R.L. (2007) Comparison of whole chloroplast genome sequences to choose noncoding regions for phylogenetic studies in angiosperms: The tortoise and the hare III. *American Journal of Botany*, **94**, 275–288

Strand, A.E., Leebens-Mack, J. & Milligan, B.G. (1997) Nuclear DNA-based markers for plant evolutionary biology. *Molecular Ecology*, **6**, 113–118.

Taberlet, P., Gielly, L., Pautou, G. & Bouvet, J. (1991) Universal primers for amplification of three noncoding regions of chloroplast DNA. *Plant Molecular Biology*, **17**, 1105–1109

Urbatsch, L.E., Baldwin, B.G. & Donoghue, M.J. (2000) Phylogeny of the coneflowers and relatives (Heliantheae: Asteraceae) based on nuclear rDNA internal transcribed spacer (ITS) sequences and chlorplast DNA restriction site data. *Systematic Botany*, **25**, 539–565.

White, T.J., Bruyns, T.D., Lee, S.B. & Taylor, J.W. (1990) Amplification and direct sequencing of fungal ribosomal RNA genes for phylogenetics. *PCR protocols: A guide to methods and applications* (ed. by M.A. Innis, D.H. Gelfand, J.J. Sninsky and T.J. White), pp. 315–324. Academic Press, San Diego, California, USA.
